# Fruit and Vegetable Intake Patterns and Their Associations with Sociodemographic Characteristics, Anthropometric Status and Nutrient Intake Profiles among Malaysian Children Aged 1–6 Years

**DOI:** 10.3390/nu9080723

**Published:** 2017-07-31

**Authors:** Kar Hau Chong, Shoo Thien Lee, Swee Ai Ng, Ilse Khouw, Bee Koon Poh

**Affiliations:** 1 Nutritional Sciences Programme, School of Healthcare Sciences, Faculty of Health Sciences, Universiti Kebangsaan Malaysia, Jalan Raja Muda Abdul Aziz, Kuala Lumpur 50300, Malaysia; karhau88@gmail.com (K.H.C.); shoothien@hotmail.com (S.T.L.); 2 FrieslandCampina, Stationsplein 4, 3818 LE Amersfoort, The Netherlands; SweeAi.Ng@frieslandcampina.com (S.A.N.); ilse.tan-khouw@frieslandcampina.com (I.K.); 3 National Co-Ordinator, the SEANUTS Study Group, Nutritional Sciences Programme, School of Healthcare Sciences, Faculty of Health Sciences, Universiti Kebangsaan Malaysia, Kuala Lumpur 50300, Malaysia

**Keywords:** fruit intake, vegetable intake, sociodemographic determinants, anthropometric status, nutrient intake, young children

## Abstract

This study aimed to assess fruit and vegetable intake patterns and their associations with sociodemographic characteristics, anthropometric status and nutrient intake profiles among Malaysian children aged 1–6 years. Using the Malaysian dataset of South East Asian Nutrition Surveys (SEANUTS Malaysia), a total of 1307 children aged 1–6 years with complete datasets were included in this analysis. Dietary intake was assessed using age-specific, validated food frequency questionnaires. On average, Malaysian children consumed 0.91 and 1.07 servings of fruits and vegetables per day, respectively. Less than one-fifth of the children achieved the daily recommended servings of fruits (11.7%) and vegetables (15.8%). Fruit intake was associated with age, parental educational level and geographical region, and vegetable intake was associated with ethnicity and geographical region. There was little evidence of an association between fruit and vegetable intake and children’s anthropometric status, but an adequate intake of fruits and vegetables contributed significantly and differently to children’s micronutrient intake. Future nutrition interventions should focus on addressing the sociodemographic determinants and be tailored to the needs of the low consumers to more effectively promote and encourage the adequate intake of fruit and vegetables among young children.

## 1. Introduction

Fruits and vegetables have been recognised as the key components of a healthy diet because of their high levels of health-promoting nutrients and bioactive compounds, including vitamins, minerals, fibres and phytochemicals [1]. A minimum daily intake of 400 g of fruits and vegetables (excluding potatoes and other starchy tubers) is recommended for the prevention of chronic diseases and micronutrient deficiencies, particularly in less developed countries [2]. More recent reviews in adult populations have concluded that the increased consumption of fruits and vegetables not only reduces the risks of obesity or weight gain [3] and various types of chronic diseases (e.g., hypertension, coronary heart disease, and stroke) [4], but is also associated with a lower risk of all-cause mortality, particularly cardiovascular mortality [5]. In children and adolescents, emerging but as yet inconclusive evidence exists for the protective effect of fruit and vegetable intake on adiposity or obesity [6,7].

Despite their well-documented health benefits, the intake of fruits and vegetables remains low worldwide, particularly among children and adolescents. For example, in the United States, approximately 60% of children and adolescents did not meet the recommended servings for fruit, despite a significant increase (12%) in total fruit intake from 2003 to 2010. For vegetable intake, 93% of children and adolescents did not meet recommendations, with total intake remaining unchanged during the same time period [8]. A similar situation was also reported for the adolescent population in Asian countries (India, Indonesia, Myanmar, Sri Lanka and Thailand), in which 76.3% reportedly consumed less than five servings of fruits and/or vegetables per day [9]. In Malaysia, the limited data available for the childhood population also reported that fruit and vegetable intakes were far below recommended levels [10,11]. Given that food intake patterns established during childhood may track into adulthood [12] and the potential associations of fruit and vegetable intake with other eating [13,14] and health behaviours (e.g., physical activity and sedentary behaviour) [9,15], increasing the intake of fruits and vegetables among children is therefore a public health priority.

To the best of our knowledge, limited data are available on fruit and vegetable intake in young children, especially among toddlers and pre-schoolers, in Asian countries and little is known about the extent to which the intake of fruit and vegetables influences their anthropometric status and dietary nutrient intake. A better understanding of these associations is particularly important for providing insights towards the development of more effective strategies and approaches to target the double burden of malnutrition occurring in many developing countries [16,17,18], including in Malaysia [19,20]. In addition, fruit and vegetable intake has been previously reported to be closely related to sociodemographic characteristics [21,22]. Identifying the associated sociodemographic determinants is thus important for designing and implementing population-specific interventions to promote and encourage fruit and vegetable intake beginning in early childhood.

Thus, the purpose of the current study was to assess fruit and vegetable intake patterns and their associations with sociodemographic characteristics, anthropometric status and nutrient intake profiles among a nationally-representative sample of Malaysian young children aged 1–6 years.

## 2. Materials and Methods

### 2.1. Study Design and Sampling

The current study analyses the Malaysian dataset of the South East Asian Nutrition Surveys (SEANUTS), a cross-sectional nutrition survey that was conducted simultaneously in Indonesia, Malaysia, Thailand and Vietnam during 2010–2011 [23]. Using stratified random sampling method, a nationally-representative sample of 3542 Malaysian children aged 0.5–12 years was recruited from nurseries, kindergartens and schools located in rural and urban areas in the six regions of the country [20]. This study was registered in the Netherlands Trial Registry (NTR2462) and was conducted according to the Guidelines of the Helsinki Declaration for Experiments in Humans. The study protocol was approved by the Research Ethics Committee of Universiti Kebangsaan Malaysia (NN-072-2009) and has been described in detail previously [20,23]. Written informed consent was obtained from the parent or guardian of all participating children.

### 2.2. Sample

A total of 1505 children (787 boys, 718 girls) aged 1–6 years participated in the Malaysian SEANUTS. Among those who had completed the food frequency questionnaires (FFQs) with valid data for food group analyses (*n* = 1423), 116 (8.1%) were found to have missing data in parental educational level and household income and were thus excluded from the current analysis. The final sample includes 1307 children (686 boys, 621 girls) with complete datasets.

### 2.3. Dietary Assessment

Two different sets of validated semi-quantitative food frequency questionnaires (FFQs) were used to assess the dietary intakes of children: FFQ1 for children aged below 2 years and FFQ2 for children aged 2–6 years. Detailed descriptions of these FFQs have been published elsewhere [20,24]. Briefly, the parents or caregivers were asked to report the frequency and portions consumed by their children for each food item listed in the FFQs during the month prior to data collection.

To quantify and summarise the intake patterns of the fruit and vegetable groups, the original portion size of each food item in these two food groups was first translated into the recommended serving sizes according to the Malaysian Dietary Guidelines [25]. One serving of fruit contains 15 g of carbohydrate, whereas one serving of vegetables equals one cup (200 mL) of raw vegetables or half cup of cooked vegetables for both dark-green leafy vegetables and fruit vegetables. The amount consumed was then determined by multiplying the conversion factor of the reported frequency with the standardised serving size of the food group. The total intake (servings/day) for the fruit and vegetable groups was then calculated and compared with the recommended number of servings in the dietary guidelines for children and adolescents [26]. Children were classified as achieving the intake recommendations if they consumed two servings of fruit or two servings of vegetables daily [26].

In the present study, the vegetable group included green leafy vegetables (e.g., lettuce, spinach and swamp cabbage), bean vegetables (e.g., long beans, lady fingers and bean sprouts), cruciferous vegetables (e.g., cabbage, broccoli and cauliflowers) and carrot/cucumber/tomato. The fruit group included apples, oranges, bananas, watermelon, papaya, grapes and Chinese pears. Additional food items, including fruit and vegetable purees, were assessed for children aged below 2 years. The mean daily energy and nutrient intakes were also calculated using a validated Excel-based platform [27] that incorporates nutrient values from the available databases [28,29] and food product labels.

### 2.4. Sociodemographic Variables

Data on sociodemographic variables, including age, sex, ethnicity, parents’ educational level and monthly household income, were collected from the parents or the caregivers using a structured questionnaire. Children were classified into four main ethnic groups, namely Malays, Chinese, Indians, and Others, which includes Sabah and Sarawak *bumiputras* and also other *bumiputras* (such as *Orang Asli*). Parental educational level was categorised into three groups: no schooling or primary education, secondary education, and tertiary education. The monthly household income data in Malaysian ringgit (RM) was also categorised into three groups: low (<RM2300), middle (RM2300–5599), and high (≥RM5600) [30].

### 2.5. Anthropometric Measurements

Body weight was measured to the nearest 0.1 kg using a calibrated digital scale (Model 803; SECA, Hamburg, Germany). For children older than 2 years, height was measured to the nearest 0.1 cm using a portable stadiometer (Model 213; SECA, Hamburg, Germany). In younger children, length was measured to the nearest 0.1 cm while lying supine using a wooden measuring board. Following the WHO STEPwise protocol [31], waist circumference (WC) was measured to the nearest 0.1 cm at the midpoint between the lowest rib and iliac crest using a Lufkin tape (Model W606PM; Apex Tool Group, Baltimore, MD, USA). All measurements were performed by trained research assistants with children in light clothing and without shoes.

Body mass index (BMI) was calculated by dividing weight by height squared (kg/m^2^). Z-scores for weight-for-age (WAZ), height-for-age (HAZ) and BMI-for-age (BAZ) were then determined using WHO Anthro version 3.1.0 (for children aged 5 years and below) (World Health Organization, Geneva, Switzerland) and WHO AnthroPlus version 1.0.3 (for children older than 5 years) (World Health Organization, Geneva, Switzerland). Stunting was defined as HAZ values ≤ −2. Thinness was defined as BAZ values ≤ −2, and overweight was defined as BAZ values ≥ 1 in children older than 5 years and ≥2 in children younger than 5 years. Cut-off values for obesity were BAZ ≥ 2 or ≥ 3 in children older or younger than 5 years, respectively [32,33].

### 2.6. Statistical Analysis

Statistical analyses were performed using the Complex Samples module of the IBM SPSS Statistics for Windows version 20.0 (IBM Corporation, New York, NY, USA), in which the data were weighted to be representative of the Malaysian childhood population using weight factors based on the 2010 Malaysian population census [34]. Population estimates of the mean and standard error (SE) are presented for continuous data, and the percentage and 95% confidence interval (CI) are presented for categorical data. Complex samples general linear model analyses with Bonferroni adjustment for multiple comparisons were used to determine the differences in the mean daily intake of fruits and vegetables (servings/day) by sociodemographic characteristics and to assess differences in anthropometric measures and dietary energy and nutrient intakes by fruit and vegetable intake patterns, adjusted for the variables as indicated in the footnote of the respective table. A complex samples logistic regression analysis was used to examine the proportion differences of children who achieved the recommended servings of fruits and vegetables comparing between different sociodemographic groups. The significance level was set at *p* < 0.05 for all analyses.

## 3. Results

The current analyses include data from 1307 children (mean age 3.9 ± 0.1 years), which represents an estimated 2.44 million Malaysian children aged 1–6 years. Table 1 presents the sociodemographic characteristics of children and their daily intakes of fruit and vegetables. On average, children consumed 0.91 servings/day of fruit, and only 11.7% achieved the recommended two servings per day. The intake of fruit was reported to be significantly higher among the oldest children (5–6 years old: 1.14 servings/day) and those whose fathers (0.98 servings/day) or mothers (1.00 servings/day) had completed secondary education. Significantly more children who achieved the fruit intake recommendation were in the oldest age group (16.2%) and in the Sarawak region (17.4%). For vegetables, the mean daily intake was 1.07 servings/day, and 15.8% of children reportedly achieved the recommended two servings per day. The Malay ethnic group (0.87 serving/day; 11.3%) and those living in Sabah (0.85 serving/day; 8.9%) both showed significantly lower mean intakes of vegetables and had smaller proportions of children who achieved the intake recommendations compared to their respective counterparts. Only 3.4% of the children consumed two servings of fruits and two servings of vegetables, and thus achieved the intake recommendations for both fruit and vegetable groups.

Table 2 shows the anthropometric characteristics of the children based on their fruit and vegetable intake patterns. Height and HAZ differed significantly between the fruit intake groups. Children who consumed fewer than the recommended two servings per day had significantly higher values (height: 98.7 vs. 97.8 cm, *p* < 0.05; HAZ: −0.63 vs. −0.85, *p* < 0.05) than those who consumed two or more servings per day, after adjusting for sociodemographic and energy intake variables. However, no significant differences were observed for other anthropometric measures or the prevalence of stunting, thinness, overweight and obesity between the children who achieved their fruit intake recommendations and the children who did not. For vegetable intake, no significant differences were observed in the anthropometric measures and the prevalence of stunting, thinness, overweight and obesity was not significantly different between the two intake groups.

Table 3 presents the dietary energy and nutrient intakes of children relative to their fruit and vegetable intake patterns. Daily energy intake was reported to be significantly higher among children who achieved their fruit (*p* < 0.001) or vegetable (*p* < 0.001) intake recommendations after adjusting for sociodemographic variables. After further adjustment for daily energy intake (in addition to sociodemographic variables), children who achieved their fruit intake recommendation were found to have significantly higher intakes of carbohydrates (*p* < 0.001) and vitamin C (*p* < 0.001), but lower intakes of fat (*p* < 0.001), thiamine (*p* < 0.05), riboflavin (*p* < 0.01), calcium (*p* < 0.05), iron (*p* < 0.05) and zinc (*p* < 0.001) compared to children who did not achieve the recommendation. On the other hand, children who achieved their vegetable intake recommendations consumed significantly more vitamin A (*p* < 0.001), vitamin C (*p* < 0.001), calcium (*p* < 0.05) and iron (*p* < 0.01), but less thiamine (*p* < 0.05) and riboflavin (*p* < 0.01) compared with those who did not.

## 4. Discussion 

This study is one of the first to provide nationally-representative data on daily intakes of fruits and vegetables in standard serving sizes according to the dietary guidelines, and their associations with sociodemographic characteristics, anthropometric status and nutrient intake profiles among Malaysian children aged 1–6 years. Overall, the children’s mean intakes of fruits and vegetables were found to be approximately only half of the recommended number of daily servings, with less than one-fifth achieving the daily fruit or vegetable intake recommendations. Fruit intake was found to be associated with age, parental educational level and geographical region, and vegetable intake was associated with ethnicity and geographical region. The intake of fruits, but not vegetables, was associated with anthropometric measures, but the association was only observed with height and HAZ variables and was likely not clinically significant. Moreover, there were no significant associations between fruit and vegetable intake and children’s stunting and body weight status. In terms of the nutrient intake profile, a daily intake of two or more servings of fruit was associated with greater intake of carbohydrates and vitamin C, while daily intake of two or more servings of vegetables was associated with higher intake of vitamin A, vitamin C, calcium and iron, regardless of total daily energy intake.

Our study revealed that age was a significant determinant for fruit intake, but not vegetable intake. The mean daily intake of fruit increased significantly across the age groups, and the percentage of children achieving intake recommendation was 2.5 times higher among the oldest age group (5–6 years: 16.3%) relative to the youngest age group (1–2 years old: 6.6%). Similar results were also noted in a previous analysis of the primary school-aged children who participated in SEANUTS Malaysia [10], whereby a significantly higher proportion of older children (10–12 years: 19.6%) were found to have achieved the dietary recommendations for fruit intake than younger children (7–9 years; 13.4%). Altogether, the results seem to suggest that Malaysian children tend to eat more fruits as they get older. This finding is, however, in contrast to previously published data from European and American populations [22,35], which have shown an age-associated decline in total fruit and vegetable intake among children and adolescents. The inter-population variation and cultural differences in food intake patterns could be a possible explanation for the inconsistency of findings from different parts of the world. Moreover, a study of urban children in Kuala Lumpur in year 2015 found that children aged 1–3 years consumed approximately 700 mL or 3.5 cups of milk per day, which exceeds the current recommendation of 2–3 cups daily (200 mL/cup), and also reported that milk alone contributed to 50–63% of their daily energy requirement [36]. Over-consumption of milk could lead to lower dietary diversity in young children, especially in terms of the intake of fruit, which is commonly perceived by the Malaysian population as “dessert” rather than as a part of main meals. It is also important to note that the recommended serving sizes for the fruit and vegetable groups in the Malaysian Dietary Guidelines are the same for all age groups [25], but younger children usually consume a smaller portion size than older children, which may explain the lower achievement rates of fruit intake recommendation among the younger age group.

Parental educational level was also found to be associated with children’s fruit intake in this study. Higher or more frequent intake of fruits and vegetables has been previously reported to be associated with higher parental educational levels in European populations [37,38]. However, our analysis showed that the mean intake of fruit was highest among children whose father or mother had completed secondary education, although the proportions of children that achieved fruit intake recommendation did not vary significantly across educational attainment levels. The lower intake of fruit among those whose mother had tertiary education could be because young children usually depend on their parents for food provision, but most of the more highly educated parents would have limited time to prepare food for their children as they are more likely to have full-time employment. Nevertheless, more studies are required to explore the factors that could possibly mediate this association.

Consistent with our previous results in the SEANUTS Malaysia primary school-aged children [10], the present findings among children aged six year and below showed that vegetable intake differed significantly between ethnic groups. Malay children showed the lowest mean daily intake of vegetables, with significantly less children achieving vegetable intake recommendation compared to Chinese and Indian children. This finding is also comparable to those reported by Nurul-Fadhilah et al. in 2016 [39], in which the healthy-based food pattern characterised by high intakes of fruits and vegetables, dairy products, nuts and cereal-based foods was significantly more common among the Chinese than the Malay adolescents in Kelantan, Malaysia. These differences in food preferences or practices may reflect the variations in socio-cultural practices or religious beliefs across the various ethnic groups in Malaysia [40]. In addition, the current study also found that fruit and vegetable intake varied significantly by geographical region, which is consistent with the findings of other national population-based surveys on the influence of the location of residence on fruit and vegetable intakes in the Malaysian adult population [41,42]. Although we speculate that this could be due to cultural differences or geographical variations in the availability of fruits and vegetables, the underlying reasons for these differences are still not well-understood and warrant further investigation.

This study provides little evidence of an association between fruit and vegetable intake and anthropometric status in children. Although children who did not achieve the fruit intake recommendation were found to be taller and to have larger HAZ values than those who did achieve the recommendation, the differences between the two groups were relatively small and were likely not clinically significant (height: 0.9 cm, HAZ: 0.22 unit). In fact, no significant differences were observed in the prevalence of stunting, thinness, overweight or obesity between the two groups, suggesting that fruit and vegetable intake may not have any significant influence on a child’s anthropometric status. This finding is comparable to those reported by Norimah et al. [43] in 2014 that found no significant association between BMI-for-age with the frequency of fruit and vegetable intake among preschoolers in Peninsular Malaysia. Nevertheless, studies conducted in Western populations have yielded mixed results [44,45,46]. Using the dataset of the Third National Health and Nutrition Examination Survey (NHANES III), Bradlee and colleagues [45] found no significant associations between the mean intakes of fruit and vegetables and central adiposity measures among children aged 5–11 years; however, the combined intake of fruit and vegetable was reported to be inversely associated with central obesity among adolescents aged 12–16 years. On the other hand, Matthews et al. [46] found that, in children and adolescents aged 6–19 years in Southern California, higher intake of vegetables, but not fruits, was associated with lower risk of being overweight. One possible reason for the mixed findings is that the large inter- and intra-population variations in food preparation methods could contribute to significant changes in the energy and nutrient content of fruits and vegetables, thus modifying their effects on anthropometric or adiposity status [6]. In addition, the overall dietary intake pattern and other sociodemographic and lifestyle variables could also partly explain the inconsistent results, given the complex and multifaceted nature of the determinants of children’s nutritional status.

Another significant finding that emerged from this study is that the nutritional contributions of fruits and vegetables vary significantly, especially with regard to intake of micronutrients. The consumption of fruit was found to generally contribute more to carbohydrate and vitamin intake, whereas the consumption of vegetables resulted in greater intake of vitamins and minerals, which lend further support to the importance of consuming a wide variety of fruits and vegetables to ensure adequate intake of different micronutrients or bioactive compounds [1]. Interestingly, children with fruit or vegetable intakes below the recommended levels showed significantly higher energy-adjusted intakes of thiamine and riboflavin than those who achieved the recommendations. We have also analysed the same data without energy-adjustment, which appear to give contradictory results [47]. The results of the energy-unadjusted analysis showed that children with an inadequate intake of fruits or vegetables had similar intake levels of thiamine and riboflavin, but lower intakes of minerals (such as calcium and iron). They were also more likely than the children who achieved fruit or vegetable intake recommendations to be at risk of inadequate intakes for most micronutrients [47]. We theorise that these differences could be because the intake of most nutrients is usually correlated with total energy intake (either through their direct contribution to energy intake or because of higher food consumption resulting in higher energy intake) [48]. Therefore, failing to account for the variations in total energy intake could possibly confound the true effect of dietary patterns (i.e., fruit and vegetable intake) on the intake of all micronutrients. Our results seem to confirm the importance of adjusting for total energy intake when conducting epidemiologic analyses of nutrient intakes.

The observed higher intakes of thiamine and riboflavin suggests the possibility that children with inadequate intakes of fruits and vegetables tend to eat more from other food groups, such as meat and poultry, which are known to be important food sources of thiamine [49] and riboflavin [50]. This hypothesis is supported by previous observations that a large majority of Malaysian children had adequate intake of the meat/poultry group, but not other food groups, including fruits and vegetables [10,11]. Excessive reliance on milk and dairy products as the primary food sources in young children [36] could also explain the higher intakes of specific micronutrients among those with low fruit and vegetable intake in the present study.

The main limitation of this study is that the dietary intake data were collected using parent or caregiver proxy-reporting method because the children involved were too young and unable to provide accurate self-reports. In this case, some misreporting of total food intake could occur since the parent or caregiver may not be able to accurately report their child’s dietary intake during the time they spent out-of-home; e.g., at preschool, kindergarten, or childcare centre. Nevertheless, the use of age-specific and validated semi-quantitative FFQs adds credibility to the findings of this study as it provides a more representative picture of the habitual dietary intake of children from different age groups compared to other assessment methods (e.g., 24-h or three-day diet recall). Next, we did not evaluate seasonal variations in the availability of fruits and vegetables in different regions of the country, which could possibly influence the intake of the population throughout the period of data collection. However, this is the first nationwide study that focuses not only on assessing the fruit and vegetable intake patterns but also on determining their associations with sociodemographic characteristics, anthropometric measures and nutrient intake profiles among young children. These data provide important background information to health professionals and policy-makers that could aid in the development of suitable and effective strategies to promote fruit and vegetable intake as part of healthy dietary habits in Malaysian children.

## 5. Conclusions

This study revealed that the majority of Malaysian children still fall short of achieving the dietary recommendations for fruit and vegetable intake. Age, parental education level and geographical region were the determinants for fruit intake, and ethnicity and geographical regions were the determinants for vegetable intake. There is little evidence of an association between fruit and vegetable intake and anthropometric status. On the other hand, adequate intakes of fruits and vegetables were found to contribute significantly but differently to children’s nutrient intake profiles, particularly micronutrient intake. These findings suggest that high intakes of fruits and vegetables may improve micronutrient intake and adequacy, but they may have little or no impact on the child’s anthropometric status. Future nutrition interventions that promote fruit and vegetable intake should address the specified sociodemographic determinants and should be tailored to the needs of the specific groups of children identified as low consumers. Multi-sectorial partnerships are also important and necessary to create supportive environments for the effective implementation of fruit and vegetable promotion initiatives across different levels, including school, community and national levels.

## Figures and Tables

**Table 1:** Sociodemographic characteristics and daily intakes of fruits and vegetables.

Characteristics	Proportion by Sociodemographic Characteristics (%)	Fruit Intake			Vegetable Intake		
		Mean	SE	% Rec. ^†^	Mean	SE	% Rec. ^†^
Overall		0.91	0.03	11.7	1.07	0.05	15.8
Sex							
Boys	50.3	0.86	0.04	10.9	1.03	0.08	14.2
Girls	49.7	0.96	0.05	12.6	1.11	0.06	17.3
Age group (years)							
1–2	34.5	0.63 ^a^	0.05	6.6 ^d^	1.03	0.11	14.8
3–4	34.8	0.97 ^b^	0.06	12.8 ^e^	1.06	0.08	15.4
5–6	30.7	1.14 ^c^	0.06	16.3 ^e^	1.13	0.08	17.4
Area of residence							
Rural	21.8	0.99	0.05	13	0.96	0.06	12.2
Urban	78.2	0.88	0.04	11.4	1.1	0.06	16.8
Ethnicity							
Malay	59.2	0.9	0.04	12.2	0.87 ^a^	0.06	11.3 ^d^
Chinese	18.9	0.86	0.07	9.1	1.47 ^b^	0.12	23.9 ^e,f^
Indian	5.6	1	0.15	13	1.86 ^a,b^	0.46	34.1 ^f^
Others	16.3	0.96	0.08	12.6	1.07 ^a,b^	0.12	16.3 ^d,e^
Geographical region							
East Coast	9.1	1	0.08	12.3 ^d,e^	0.95 ^a,b,c^	0.12	14.8 ^d,e^
Southern	29.1	0.9	0.06	11.7 ^d,e^	1.15 ^a,b^	0.09	17.7 ^d^
Northern	16.3	0.92	0.09	12.2 ^d,e^	1.51 ^a^	0.2	22.3 ^d^
Central	21.7	0.78	0.07	10.9 ^d,e^	0.87 ^b,c^	0.11	12.8 ^d,e^
Sarawak	9.8	1.05	0.1	17.4 ^d^	0.99 ^a,b,c^	0.12	17.3 ^d^
Sabah	14	0.94	0.07	8.2 ^e^	0.85 ^c^	0.06	8.9 ^e^
Paternal education							
No schooling/Primary education	5	0.68 ^a^	0.1	6.7	1.03	0.32	17.5
Secondary education	56	0.98 ^b^	0.04	12.5	1.05	0.07	14.8
Tertiary education	39	0.83 ^a,b^	0.05	11.2	1.11	0.08	16.9
Maternal education							
No schooling/Primary education	5.5	0.73 ^a,b^	0.12	5.4	1.28	0.3	26.6
Secondary education	52.1	1.00 ^a^	0.04	13.3	1	0.07	14.9
Tertiary education	42.4	0.82 ^b^	0.05	10.6	1.13	0.08	15.4
Monthly household income							
Low (<RM2300)	37.2	0.95	0.05	11.3	1.07	0.09	17.8
Middle (RM2300–5599)	43.2	0.91	0.05	12.9	1	0.08	13
High (≥RM5600)	19.6	0.82	0.07	9.8	1.24	0.12	18.1

Abbreviation: RM, Malaysian Ringgit (RM 1 = 0.23 US $ as of 15 May 2017). **^†^** Percentage of children achieving fruit and vegetable intake recommendations (rec.): 2 servings of fruits and 2 servings of vegetables daily (NCCFN 2013). ^a,b,c^ Values that do not share the same alphabets were significantly different from each other within the sociodemographic group based on complex sample general liner model analysis at *p* < 0.05. ^d,e,f^ Values that do not share the same alphabets were significantly different from each other within the sociodemographic based on complex sample logistic regression analysis at *p* < 0.05.

**Table 2:** Anthropometric status relative to daily fruit and vegetable intake patterns.

	Fruit Intake				Vegetable Intake			
	<2 Servings/Day		≥2 Servings/Day		<2 Servings/Day		≥2 Servings/Day	
	Mean	SE	Mean	SE	Mean	SE	Mean	SE
Weight (kg)	15.6	0.1	15.1	0.3	15.6	0.1	15.5	0.2
Height (cm)	98.7 *	0.2	97.8	0.5	98.6	0.2	98.8	0.4
WC (cm)	48.8	0.3	48.2	0.5	48.8	0.3	48.6	0.4
BMI (kg/m^2^)	15.7	0.1	15.6	0.2	15.7	0.10	15.6	0.2
BAZ	0.04	0.07	0.03	0.14	0.05	0.08	−0.03	0.13
WAZ	−0.34	0.06	−0.44	0.12	−0.35	0.06	−0.39	0.12
HAZ	−0.63 *	0.05	−0.85	0.11	−0.66	0.05	−0.62	0.10
Prevalence of (%):								
Stunting	10.8		12.5		11.1		10.5	
Thinness	3.4		2.3		3.3		2.8	
Overweight	5		8.4		5.2		6.3	
Obesity	4.4		2.4		4.1		4.5	
Overweight/Obesity	9.4		10.8		9.3		10.8	

Abbreviations: WC, waist circumference; BMI, body mass index; BAZ, BMI-for-age Z-score; WAZ, weight-for-age Z-score; HAZ, height-for-age Z-score. Analyses were adjusted for age, sex, ethnicity, area of residence and daily energy intake. Significantly higher than the comparison group within each food group based on complex sample general linear model analysis at * *p* < 0.05.

**Table 3:** Dietary energy and nutrient intakes relative to daily fruit and vegetable intake patterns.

	Fruit Intake				Vegetable Intake			
	<2 Servings/Day		≥2 Servings/Day		<2 Servings/Day		≥2 Servings/Day	
	Mean	SE	Mean	SE	Mean	SE	Mean	SE
Energy (kcal) ^†^	1242	17	1487 ***	32	1243	17	1386 ***	29
Carbohydrate (g) ^‡^	169.8	0.7	178.9 ***	1.7	171.3	0.8	169.3	1.6
Protein (g) ^‡^	47.4	0.4	46.0	0.7	47.0	0.4	48.1	0.8
Fat (g) ^‡^	46.5 ***	0.7	42.3	1.0	46.3	0.8	44.9	0.7
Vitamin A (μg) ^‡^	898	25	997	48	873	27	1056 ***	35
Vitamin C (mg) ^‡^	84	2	122 ***	4	82	1	113 ***	3
Thiamine (mg) ^‡^	1.7 *	0.1	1.4	0.1	1.8 *	0.1	1.3	0.1
Riboflavin (mg) ^‡^	2.2 **	0.1	1.7	0.1	2.3 **	0.1	1.7	0.1
Niacin (mg) ^‡^	15.0	0.2	15.4	0.4	14.9	0.2	15.4	0.4
Calcium (mg) ^‡^	697 *	10	643	22	682	10	726 *	18
Iron (mg) ^‡^	13.3 *	0.1	12.7	0.3	13.1	0.1	13.8 **	0.2
Zinc (mg) ^‡^	7.6 ***	0.1	6.6	0.2	7.4	0.1	7.7	0.2

^†^ Analyses were adjusted for age, sex, ethnicity, area of residence and monthly household income. ^‡^ Analyses were adjusted for age, sex, ethnicity, area of residence, monthly household income and daily energy intake. Significantly higher than the comparison group within each food group based on complex sample general linear model analysis at * *p* < 0.05, ** *p* < 0.01 and *** *p* < 0.001.
